# Live or Die: Choice Mechanisms in Stressed Cells

**DOI:** 10.1155/2015/454863

**Published:** 2015-10-08

**Authors:** Francesco Cecconi, Laura Soucek, Dennis D. Taub, Elio Ziparo

**Affiliations:** ^1^Cell Stress and Survival Unit, Danish Cancer Society Research Center, Strandboulevarden 49, 2100 Copenhagen, Denmark; ^2^IRCCS Fondazione Santa Lucia, 00143 Rome, Italy; ^3^Department of Biology, University of Rome “Tor Vergata”, 00133 Rome, Italy; ^4^Vall d'Hebron Institute of Oncology (VHIO), 08035 Barcelona, Spain; ^5^Department of Biochemistry and Molecular Biology, Universitat Autònoma de Barcelona, 08193 Bellaterra, Spain; ^6^Institució Catalana de Recerca i Estudis Avançats (ICREA), 08010 Barcelona, Spain; ^7^Center for Translational Studies, Medical Services, VA Medical Center, Department of Veteran Affairs, Washington, DC 20422, USA; ^8^Department of Anatomy, Histology, Forensic Medicine and Orthopedics, Section of Histology and Medical Embryology, Sapienza University of Rome, 00161 Rome, Italy

Cells respond actively to the environmental changes, constantly adjusting their structure and function in order to maintain cellular homeostasis. Such changes consequent to both physiological stresses and/or pathological stimuli may be modulated in terms of type, degree, and duration and induce multiple cellular responses. Several different signaling pathways can in fact lead to repair, adaptation, or, in some cases, cell death. A prolonged inflammatory response may ultimately lead to several pathological conditions, including cancer. The specific cell response under stressful conditions has therefore crucial effects not only on the single cell fate but also more generally on tissue homeostasis and ultimately on organism health. This special issue, analyzing different experimental models, aims at highlighting some of the key mechanisms involved in cellular stress response.

Topics of this issue include different stressful conditions such as* oxidative*,* hypoxic*,* endoplasmic reticulum (ER)* and* inflammatory stress* and consequent cell responses such as* apoptosis*,* necroptosis*,* cell cycle arrest*,* mitotic cell death*,* pyroptosis*, and* survival*.

Among different types of stress,* oxidative stress* is causally implicated in degenerative diseases onset. In particular nitrooxidative stress has been reported to be implicated in the loss of specific neuronal populations in Parkinson's disease and amyotrophic lateral sclerosis. In the present special issue, the manuscript entitled “S-Nitrosoglutathione Reductase Plays Opposite Roles in SH-SY5Y Models of Parkinson's Disease and Amyotrophic Lateral Sclerosis” by* S. Rizza* and collaborators demonstrates that denitrosylating enzyme S-nitrosoglutathione reductase (GSNOR) modulation has different effects on neuronal viability depending on the applied stimulus and highlights GSNOR as a crucial molecular player of neuronal homeostasis. The review entitled “Redox Signaling in Diabetic Nephropathy: Hypertrophy versus Death Choices in Mesangial Cells and Podocytes” by* G. Manda* and collaborators emphasizes the role of the* oxidative stress* in diabetic nephropathy, which may act as trigger, modulator, and linker within the complex network of pathologic events. This review particularly addresses the molecular switches deciding on the renal cells fate in diabetic nephropathy leading to hypertrophy versus death choices in mesangial cells and podocytes.

On the other hand, amyotrophic lateral sclerosis is a multifactorial disease caused by motor neuron degeneration and is often associated with mutation of the* superoxide dismutase 1 (SOD1)* gene (encoding an enzyme that uses copper and zinc to break down toxic, charged oxygen molecules called superoxide radicals), thus affecting SOD1 antioxidant activity. The most studied* SOD1* mutation is the G93A substitution. The manuscript entitled “Postmitotic Expression of SOD1^G93A^ Gene Affects the Identity of Myogenic Cells and Inhibits Myoblasts Differentiation” by* M. Martini* and collaborators demonstrates that* oxidative stress* associated with SOD1^G93A^ expression in C2C12 myogenic cells inhibits their myogenic program and promotes a fibroadipogenic phenotype.

*Hypoxic stress* plays a key role in heart disease, cancer, stroke, and other causes of mortality. The review entitled “Hypoxia Inducible Factor Pathway and Physiological Adaptation: A Cell Survival Pathway?” by* H. Kumar* and D.-K. Choi describes the role of the Hypoxia Inducible Factors (HIFs) in the* survival pathways* activated to regulate cellular adaptation to hypoxia.

Hypoxia and other environmental factors such as nutrient deprivation, pH changes, and reduced vascularization contribute instead to endoplasmic reticulum stress (*ER stress*) and activate the Unfolded Protein Response (UPR) in tumor cells. The review “Cancer Microenvironment and Endoplasmic Reticulum Stress Response” by* C. Giampietri* and collaborators describes the molecular mechanisms of UPR and their role in promoting cancer cells survival and proliferation.

Drug Induced Liver Injury (DILI) represents an adverse drug reaction leading to severe liver damage. Kupffer cells sense hepatic stress and produce cytokines thus stimulating an immune response. In the manuscript entitled “Subtoxic Concentrations of Hepatotoxic Drugs Lead to Kupffer Cell Activation in a Human* In Vitro* Liver Model: An Approach to Study DILI”* V. Kegel* and collaborators describe a human Kupffer cell culture model* in vitro* for the investigation of immune-mediated signaling in hepatic* inflammatory stress* involved in the pathogenesis of DILI.

Long-standing* inflammatory stress* may also promote tumor development, growth, and progression. In the manuscript entitled “Anti-Inflammatory Effects of a Methanol Extract from the Marine Sponge* Geodia cydonium* on the Human Breast Cancer MCF-7 Cell Line”* S. Costantini* and collaborators demonstrate that a methanol extract from this marine sponge reduces levels of VEGF and exerts a dose-dependent anti-inflammatory effect.

In the manuscript entitled “Multidrug Resistance Protein-4 Influences Aspirin Toxicity in Human Cell Line,”* I. Massimi* and collaborators investigate aspirin effect on the efflux transporter protein Multidrug Resistance Protein-4 (MRP4) expression in HEK-293 cells. The authors demonstrate that low nontoxic aspirin dosages may lead to MRP-4 overexpression, thus increasing cellular detoxification of aspirin. Since overexpression of efflux transporters in human cells is a mechanism of resistance to drugs, the manuscript by* I. Massimi* et al. contributes to a better comprehension of molecular mechanisms adopted by cancer cells for* survival* in stressful conditions during chemotherapy.

Various pathological processes such as ischemic brain injury, myocardial infarction, organ transplantation, and virus replication are accompanied by strong inflammatory stress and* necroptosis*. The manuscript entitled “Necroptotic Cell Death Signaling and Execution Pathway: Lessons from Knockout Mice” by* J. Belizário* and collaborators describes recent discoveries regarding necroptosis execution pathway. The authors outline and discuss important phenotypes of knockout mice models that serve to define the role of* CASPASE 8*,* FLIP*, and* FADD* genes and other major components of necroptotic signaling.

In parallel,* pyroptosis* is a recently identified type of regulated cell death associated with inflammatory response and with features distinct from both apoptosis and necroptosis. Its role in cardiovascular diseases is addressed in the manuscript “Looking for Pyroptosis-Modulating miRNAs as a Therapeutic Target for Improving Myocardium Survival” by* S. Lee* et al. The authors aim at elucidating the possible role of miRNAs as therapeutic targets for preventing excessive myocardial pyroptosis in cardiovascular diseases.

Progression through the cell cycle is one of the most important decisions during the life of a cell and several kinds of stress are able to influence this choice. The manuscript “Cellular Response upon Stress: p57 Contribution to the Final Outcome” by* M. N. Rossi* and* F. Antonangeli* focuses on the contribution of cyclin-dependent kinase inhibitor p57 in regulating* cell cycle arrest* and* apoptosis* after cellular stress with particular attention to cancer cells.

Finally, the review entitled “Targeting the mitotic catastrophe signaling pathway in cancer” by* M. M. Mc Gee* describes the molecular mechanism of* mitotic cell death*, a mechanism to remove defective and genomically unstable cells. Mitotic catastrophe is a regulated antiproliferative process that occurs as a consequence of defective mitosis and may precede apoptosis, thus representing a further response under stressful conditions.

The readers will find in this special issue not only interesting data and updated reviews on possible mechanisms of cellular stress response, but also relevant questions waiting to be answered. The study of multiple pathways activated under stressful conditions has become increasingly complex, requiring expertise from all fields of biology. A better comprehension of such pathways might help elucidation of disease pathogenesis and may be of crucial interest in order to develop new therapeutic strategies.

*Francesco Cecconi* *Francesco Cecconi* *Laura Soucek* *Laura Soucek* *Dennis D. Taub* *Dennis D. Taub* *Elio Ziparo* *Elio Ziparo*

